# A qualitative study on gender inequality and gender-based violence in Nepal

**DOI:** 10.1186/s12889-022-14389-x

**Published:** 2022-11-01

**Authors:** Pranab Dahal, Sunil Kumar Joshi, Katarina Swahnberg

**Affiliations:** 1 Department of Health and Caring Science, Linnaeus University, 391 82 Kalmar, Sweden; 2 Department of Community Medicine, Kathmandu Medical College, 446 00 Kathmandu, Nepal

**Keywords:** Gender, Inequality, Violence, Power play, Constructivist grounded theory, Nepal

## Abstract

**Background:**

Gender inequality and violence are not mutually exclusive phenomena but complex loops affecting each other. Women in Nepal face several inequalities and violence. The causes are diverse, but most of these results are due to socially assigned lower positioning of women. The hierarchies based on power make women face subordination and violence in Nepal. The study aims to explore participants' understanding and experience to identify the status of inequality for women and how violence emerges as one of its consequences. Furthermore, it explores the causes of sex trafficking as an example of an outcome of inequality and violence.

**Method:**

The study formulated separate male and female groups using a purposive sampling method. The study used a multistage focus group discussion, where the same groups met at different intervals. Six focus group discussions, three times each with male and female groups, were conducted in a year. Thirty-six individuals, including sixteen males and twenty females, were involved in the discussions. The study used constructivist grounded theory for the data analysis.

**Results:**

The study participants identify that a power play between men and women reinforce inequality and increases the likelihood of violence for women. The findings suggest that the subjugation of women occurs due to practices based on gender differences, constricted life opportunities, and internalization of constructed differences among women. The study identifies that interpersonal and socio-cultural violence can result due to established differences between men and women. Sex trafficking, as an example of the outcome of inequality and violence, occurs due to the disadvantageous position of women compounded by poverty and illiteracy. The study has developed a concept of power-play which is identified as a cause and consequence of women's subordination and violence. This power play is found operative at various levels with social approval for men to use violence and maintain/produce inequality.

**Conclusion:**

The theoretical concept of power play shows that there are inequitable power relations between men and women. The male-centric socio-cultural norms and practices have endowed men with privilege, power, and an opportunity to exploit women. This lowers the status of women and the power-play help to produce and sustain inequality. The power-play exposes women to violence and manifests itself as one of the worst expressions used by men.

## Background

Violence against women is identified as an attempt by men to maintain power and control over women [1] and is manifested as a form of structural inequality. This structural inequality is apparent with greater agency among men [2]. The differences between sexes are exhibited in the attainment of education and professional jobs, ownership of assets, the feminization of poverty, etc., and these differences increase the risk of violence towards women [3]. The global estimate identifies that thirty percent of women experience physical and/or sexual violence during their lifetime, illustrating the enormity of this problem [4]. From a feminist perspective, lending ideas of patriarchy [5] and gender performativity [6], the understanding of gender roles prescribed by male-dominated social structures and processes helps further explore the violence and abuse faced by women [7]. According to Heise [8], men who adhere to traditional, rigid, and misogynistic views on gender norms, attitudes, and behaviors are more likely to use violence towards women. The individual and collective attitudes of men toward different established gender norms, and their reproduction explain men’s use of violence toward women [9]. It is known that gender norms influence violence, but at the same time violence also directs and dictates gender performance with fear, sanction, and corrective measures for enacting respective prescribed gender functions [10].

It is difficult for women subjected to violence to enjoy legitimate rights, as most of the infringement of their rights and violence takes place inside a private sphere of the home [11, 12]. Violence against women is the major cause of death and disability for women [13] and globally a major public health concern [14]. Establishing gender equality is fundamental for fostering justice and attaining sustainable development [15]; moreover, violence against women has to be acknowledged as a fundamental abuse of human rights [16]. A report on global violence has identified that violence against women exists at all levels of the family, community, and state. The report recommended the development of frameworks for respecting, protecting, and fulfilling women’s rights [17]. Fifteen years later, a review of the same identifies that violence continues with impunity, reaffirming violence as a major obstacle to the attainment of justice [18].

The inclusion of the gender lens to violence against women has provided more contextual evidence to explore these processes of violence. This requires the identification of unequal power relationships and an inquiry into the differences-producing various gender stereotypes [19]. This analysis of violence requires an understanding of behaviors that promote women’s subordination and factors that favor men to sustain these malpractices [8]. A closer look at the male-centric structural arrangements embedded in the social, political, and economic organization of life reveals that these structures provide lesser access and lower accountability toward women, promote systemic subordination, and create hierarchies, resulting in the increase of violence against women [20]. This unequal gender power relationship reinforced and manifested by social approval of men’s authority over women is found operative at multiple levels and helps to produce diversities of inequalities and violence [21, 22].

The inequalities faced by women in Nepal majorly stem from socio-cultural, economic, and religious factors and influencers that define traditional roles and responsibilities between men and women [23]. The inequalities are more evident and pronounced in settings exhibiting prominent patriarchal norms restricting advantages and opportunities for the majority of women [24]. Women in Nepal are restricted inside their homes, have lesser access to life opportunities, and have limited or no involvement in decision-making on important issues directly affecting their lives [25, 26]. Figures indicative of women’s inequalities in Nepal suggest that one-third of women have no education, fifty-two percent of women are involved in non-paid jobs, and women are less likely than men to own a home or land [27]. The men in Nepalese society are positioned higher and are expected to be the breadwinner and protectors of their families. Most of these men intend to earn respect and obedience from women and are socially expected to discipline women to achieve it [28]. Many societies across the world including Nepal, recognizes violence as a private affair requiring discussion only within a family. This has led to a serious underreporting of violence committed toward women in Nepal [29]. The national gender data in Nepal is scarce, the available Nepal Demographic Health Survey 2016 identifies that since the age of fifteen, twenty-two percent of women and seven percent of women experience physical and sexual violence, respectively in the past twelve months [27].

The contributing factors for violence against women in Nepal include the lower social status of women, illiteracy, economic dependency, patriarchal society, sex trafficking, alcohol-related abuse, dowry-related violence, infidelity, extramarital affairs of husband, unemployment, and denial of sex with husband [30–32]. Nepalese women have been repressing violence with silence due to the fear of breaking relationships, receiving less love and affection from family, fear of social norms by going against men, lack of faith in the justice system, and the threat of increased violence [33]. Women and girls in Nepal are sex trafficked to various countries. Sex trafficking in Nepal is prevalent due to persistent gender inequality, violence, stigma, and discriminatory socio-cultural structures; however, the actual extent of sex trafficking is still undetermined [17, 34, 35].

The recent trends in Nepal with the increasing number of out-migration of men for employment have provided women with temporary autonomy, and a shift in the gender roles. Earlier research has identified that migration of male spouses has provided a resistance to the power dynamics for women on the other hand it has limited their mobility, required them to share decision-making with household structures, face continued social vigilance on the money received from remittance, and get central attention with their personal sexual lives [36, 37].

Morang district lies in the eastern region of Nepal. A district profile report based on a census survey [38] identifies that the place is inhabited by a close to a million population, out of which ethnic groups ( close to forty percent) live in the district with a majority (seventy-eight percent) of its population living in the rural areas. *Tharu* an ethnic group is one of the dominant population in the study area and all study participants for this study were from same *Tharu* population. A close to thirty-six percent of women in the district are illiterate and the average age of marriage is eighteen years. The report identifies that only twenty-three percent of women engage in economic activities apart from agricultural work and less than fourteen percent of women head the household. Almost eighty percent of the population in the district practice Hinduism.

This study is a part of a large intervention project and it was focused to establish a qualitative baseline of the gender status in the study area. This study aimed to explore participants’ experiences and understanding of gender inequality, violence against women, and information on sex trafficking in the Morang district of eastern Nepal. The selection of sex trafficking topic was motivated to assess the respondents’ general understanding of one of the consequences of inequality and violence faced by women. The study focused to explore factors that help to produce and sustain the practice of gender inequality and violence against women in the local community.

## Methods

### Participants

This study was part of a larger control-comparison project that used Forum Theatre interventions to promote gender equality, reduce violence against women, and increase awareness of sex trafficking [39, 40]. The participants for the focus group discussion included the intervention population from one of the randomly sampled intervention sites. A multistage focus group discussion [41] was used involving the same participants discussing various emerging topics at different periods. The participants were recruited voluntarily during an earlier quantitative data collection for the project. The study used a purposive sampling method for the selection of participants. The local field staff at the study site facilitated the recruitment of the participants. The study formulated separate male and female groups. A total of six focus groups, three each with male and female groups were conducted over twelve months. Two inclusion criteria were set for participation. First, the participants had to be part of the population of the larger study. Secondly, they had to witness and/or participate in the Forum Theatre interventions conducted in between the study. The set inclusion criteria served a dual purpose of understanding the causes of inequality and violence and further helped to develop and determine the efficacy of participatory Forum Theater intervention for awareness-raising among the study intervention groups [39].

A total of thirty-six participants consisting of sixteen males and twenty females joined the discussions. The first discussion consisted of eight participants each from groups while the second and the third discussion missed two female and four male participants respectively. The majority of the participants were 20–29 years old. *Tharu,* an ethnic community of Nepal, is a dominant population in the study area, and all the participants belonged to the same *Tharu* community. Only one female participant was unmarried, and a single married male participated in the discussions. All participants were literate, with four males completing a bachelor's level of education. Seven female participants had education below the high school level. The nuclear family with parents and their children was the major family type identified in both male and female groups. Table 1 provides the detail of the participants.

**Table 1:** Participant details

Background	Female	Male
**Number**		
1^st^ FGD	8	8
**2**^**nd**^** FGD**	6	4
**3**^**rd**^** FGD**	6	4
**Age**		
*20–29*	*6*	*7*
*30–39*	*2*	
> *40*		*1*
**Ethnicity**		
*Tharu*	8	8
**Marital Status**		
*Single*	7	1
*Married*	1	7
**Education**		
*Below high school*	7	4
* Bachelor*	1	4
**Family Type**		
*Nuclear*	5	6
*Joint*	3	2
**Family size**		
*2–4*	5	4
*5–8*	3	4

### Procedure

The focus group discussions were conducted in January 2017, April–May 2017, and January 2018. The discussions were conducted in a place recommended by the participants. An isolated place in an open setting at the premise of a local temple was used for conducting all discussions. The participants were briefed about the objectives of the discussion and written consent was obtained for their participation. Verbal consent was taken for the audio recording of the discussions. Each participant was assigned a unique numerical code before the discussions to ensure anonymity during recording, note-taking, and analysis. The discussions averaged ninety minutes during each session. The discussions were conducted with the same participants and no new participants were added during the follow-ups. A single male and female participant were missing in the second follow up and two male participants missed the final follow-up. The reason for missing participants was due to their unavailability as they were out of the village due to personal reasons.

The discussions were conducted in the Nepali language. The first author moderated all six discussions, a support field staff member took the notes, and the last author observed the discussions. The audio recordings were translated into English, and the transcriptions were checked with the recordings to verify accuracy. The field and the discussion notes were used during various stages of data analysis. The notes provided information on the discussion setting, as well as the verbal and nonverbal expressions of the participants. The notes helped to assess the impressions, emphasis, and feelings of the participants during the discussions.

### Material

The discussions used pre-formulated discussion guides with open-ended questions on inequalities, gender practices, violence, and sex trafficking. The guiding questions were based on the theoretical premise of discrimination, patriarchy, oppression, hegemony, and participation of women. Three separate discussion guides were developed for each of discussions. The guides were developed by the first and last authors. Probing was done on several occasions during the discussion to gain more clarity on the issue. Cross-checking among the participants and between the groups was done to triangulate received information. Any topic deemed appropriate for discussions and/or any unclear issues identified during the initial data analysis came up subsequently in the discussion guide during the follow-ups.

### Data analysis

This study used the constructivist grounded theory method. This method adheres to a constructivist philosophical approach wherein both researchers and participants mutually co-construct the meaning of a phenomenon [42]. This interaction is important since it helps to impart the meaning of shared experiences [42]. The constructivist grounded theory made it possible to (re) discover gender issues, important for both the researcher and the study participants. This method allowed the study to progress with responsiveness to emerging issues with an in-depth exploration of the identified issues. This clarity was achieved through repeated interactive discussions, analysis of explanations, and sharing of emergent findings with the study participants.

The audio recordings were translated and transcribed into English. Six transcripts from discussions were initially analyzed using a line-by-line coding process. The coding process helped with the fragmentation of data through interactive comparisons. Fifty-two initial codes such as gender differences, restricting women, alcohol-related violence, underreporting of sexual violence, coping, etc. were identified. The later stage of focused coding helped to achieve categorized data, providing logical sense to the developed initial codes. Three focused codes, namely, the subjugation of women, violence, and chasing dreams were formulated during the analysis. The abductive reasoning from the codes, memos, and discussion notes helped to develop the theoretical concept. The development of conceptual abstraction involved an iterative comparison of the data, codes, categories, memos, and discussion notes.

The constant communication between the authors during the stages of data analysis such as the formulation of codes, explanations of concepts, and categories helped to refine the analysis. The shared experiences of the participants and the description of the data collection and analysis included substantial details, enabling comparisons for future research and application to other similar contexts. The reliability of the study is warranted by the theoretical saturation [42] achieved by this study. This is supported by prolonged engagement with the study participants with communication on the emerging findings, and triangulation.

Reflexivity has a greater significance for the constructivist approach. The first and the second author of Nepalese origin were aware of the socio-cultural norms, stereotypes, values, and stigmas associated with gender in the local context. This helped the study to ascertain the depth of inquiry within the acceptable local normative limits. The non-Nepalese author, familiar with the study participants and Nepalese contexts, witnessed the discussions as an observer. The prior knowledge of the authors helped to critically assess different schemas, perspectives, and explanations shared by the participants. The universality of gender inequality and violence against women and its re-examination in the local context helped the authors to build upon existing knowledge by providing contextual explanations. The diversities among the authors and research participants established a basis for co-creating the perceived and observed realities.

## Findings

The section below describes the participants’ perceptions and understanding of inequality and violence. The section contains subheadings that were derived as themes in the data analysis. The first theme subjugation of women; discusses how norms, beliefs, and practices produce inferior status and positions for women. The second theme domestic and gender violence; provides a narrative of interpersonal and socio-cultural violence present in the study area. The theme of chasing dreams; discusses the process of sex trafficking as an outcome of violence. The theoretically abstracted concept of power-play identifies the cause for the generation of power imbalance producing inequality and the use of violence by men.

### Subjugation of women

The subjugation of women reflected practices and beliefs imparting positional differences for women and their social situation compared to men. The participants shared a common understanding that belief systems adhering to male supremacy have positioned women in a lower status. They provided examples of social practices of male supremacy such as males being considered as the carrier of a family name, legacy, and heritage, while women were referred to as someone else’s property. The socialization of the idea that girls will be married off to a husband and relocate themselves to their homes was identified as the major reason for instilling and perpetuating early gender differences. The participants mentioned that discriminatory practices and seclusion have situated women at the bottom rung of the gender hierarchy, establishing them as socially incompetent individuals or groups. Moreover, they inferred that selective preferences provided preparatory grounds for inequalities, and they remain attached to women throughout their lives. The participants provided examples of unequal access to education and life opportunities as a practice of selective preferences occurring in the community. They mentioned that socialization with these discriminatory beliefs and their practice helped to develop specialized gender roles from an early age. The participants provided an example of how gender intersected with mobility and resource generation in the community, it was clear from the discussions that this has restricted women inside homes but provided freedom and opportunities for men. A female participant expressed,

*A woman from a poor family is more than willing to work and support her family. But she is not allowed by the men in the family to work outside of the home.*

The participants informed that differences between the sexes were visible for women from a young age. Sharing practical examples from the community, the participants from both groups stated that girls received education mostly in low-cost government and community schools, while boys were enrolled in expensive private schools. They raised concerns that this selective investment for education, cited as the ‘building block of life’ by the participants, installed lesser capacity, and negotiating abilities in girls. A female participant stated,

*There are differences in educational opportunities for boys and girls in our community. Family provides more support for a boy’s education by enrolling him in private schools, while a girl mostly gets her education in a community school together with engagement in household work.*

The discussions revealed that women required several male anchors for their survival during their various stages of life. The participants provided examples of the shift of anchors for women which traversed from a father to a husband during marriage and later to the male child during her old age. They believed that this tradition of transferring women’s identity established men as a higher social category and stripped women of their individuality and identity. A male participant added,

*Women have to remain dependent on men throughout their lives, first with their fathers and later with their husbands. They remain completely dependent as they are not economically active. This makes men believe that they have higher authority.*

The female participants provided an example of marriage to illustrate how someone else’s decision-making had been affecting women’s lives. A participant explained that women were held responsible for household activities after marriage and any support for career progression or education was restricted despite her desire for its continuation. It was inferred that women had to drop their hopes and aspirations as the husband and his family made decisions for them. The female participants agreed that this continuous exposure to the ideas of male supremacy makes them start to believe and internalize the idea that women have lesser cognitive abilities and intelligence compared to men. A female participant stated,

*Men and women certainly have different mental abilities. Men think and act differently often in a smart way compared to women.*

The participants from both groups expressed that youth in the community were developing flexible attitudes and beliefs towards gender roles and responsibilities. They agreed that both young men and women were observed altering their roles and responsibilities shifting from traditional gender ideologies. The participants expressed that instilling these fluidity and flexible approaches in the older generation was impossible as they strictly followed traditional beliefs and practices. Few of the female participants admitted that at times young women also fail to accommodate the situation and reap benefits from available opportunities. The discussions revealed that a few of the women in the community received opportunities for independence and economic empowerment. These women had received entrepreneurial training and various skill development activities for sustaining livelihoods with practical skill-based training in tailoring, beautician, and doll-making. The female participants expressed that opportunities for independence and growth slipped away from them due to a lack of family support, financial constraints, and self-passivity. They explained that starting a business required approval from a family which was difficult to obtain. Moreover, if women made a self-decision to start up on their own, they lacked the initial capital and had to rely on men for obtaining resources. The participants further explained that the denial of men to support women were majorly due to the fear that norms of staying indoors for women will be breached and economic independence may enable women to have a similar financial footing as men. The participants stated that self-passivity in women emerged due to their engagement in household multiple roles, dependency upon males, and lack of decision-making power and abilities. A female participant summed it up by stating,

*Some of us women in the community have received entrepreneurial skills training, but we have not been able to use our skills for our growth and development. Once the training finishes, we get back to our household chores and taking care of the children.*

The female participants admitted that acceptance of belief systems requiring women to be docile, unseen, and unheard were the reasons for this self-passivity. The female participants resonated that the external controlling and unfavorable environment influenced by practices of discriminatory norms and beliefs developed self-passivity for women. A female participant expressed the cause and consequence of self-passivity as,

*Women have inhibitions to speaking their minds; something stops us from making our position clear, making us lose all the time.*

The discussions identified that gender norms were deeply engraved in various social interactions and daily life, and any deviance received strict criticism. The participants shared common examples of sanctions for women based on rigid norms like restrictive movements for women, social gossiping when women communicated with outsider men, prohibition for opinion giving in public, and lesser involvement during key decision-making at home. The participants shared that norms dictating gender roles were in place for both men and women with social sanctions and approval for their performance. A male discussion participant who occasionally got involved with cooking which was a so-called “women’s job” faced outright disapproval from his female relatives and neighbors. The male participant stated,

*If I cook or get engaged in any household jobs, it is mostly females from the home and neighborhood who make fun of me and remind me that I am a man and that I should not be doing a woman’s job.*

The foreign migration of youth looking for job opportunities has affected the *Tharu* community. It was known that a large number of men were absent from the community. The participants stated that women in such households with absent men had gained authority and control over resources, moreover, these women have been taking some of the men’s roles. The participants disclosed that these women had greater access and control over resources and were involved in the key decision-making positioning them in a relatively higher position compared to other women. It was known that this higher position for women came with a price, they were under higher social vigilance and at higher risk of abuse and violence due to the absence of ‘protective men’. It was known that women's foreign employment was associated with myths and sexist remarks. The participants shared that women had to face strict social criticisms and that their plans for livelihood and independence were related to an issue of sexual immorality and chastity. The participants from both groups strictly opposed the norms that associated women with sexual immorality but lamented that it continues. A male participant provided an insight into the social remarks received by women if she dares to go for foreign employment,

*If a woman wants to go for a foreign job, she is considered to be of loose character. The idea that she is corrupt and will get involved in bad work will be her first impression of anyone.*

Although the participant did not explicitly describe what bad work referred to as but it was inferred that he was relating it to sex work.

### Domestic and gender violence

The participants identified violence as control, coercion, and use of force against someone will occurring due to unequal status. They primarily identified men as the perpetrators and women as the victims of violence. They explained that two types of violence were observed in the community. The first type occurred in an interpersonal relationship identified as physical, emotional, and sexual violence. The second type, as explained by the participants had its roots in socio-cultural belief systems. They provided examples of dowry exchange and witchcraft accusations for the latter type. The participants identified women as primary victims and listed both men and women as the perpetrators of both types of violence. They reported that physical violence against women by men under the influence of alcohol was the most commonly occurring violence in the community. The participants from both groups confirmed that wife-beating, verbal abuse, and quarrel frequently occurred in the community. It was known from discussions that alcohol consumption among men was widespread, and its cultural acceptance was also increasing episodes of violence. One of the female participants clarified further,

*The most common violence occurring in our society is wife-beating by a husband under the influence of alcohol. We see it every day.*

The participants reported the occurrence of sexual violence in the community but also pointed out that people refrained from discussing it considering it a taboo and private affair. The participants had hesitation to discuss freely on sexual violence. During the discussions, participants from both groups informed only of rape and attempted rape of women by men as sexual violence present in the community. Despite repeated probing, on several occasions, none of the participants from either group brought up issues and discussions about any other forms of sexual violence. Participants from both groups confirmed that stories about incidents of rape or attempted rape emerged only after cases were registered with the local police. The participants presumed that incidents of rape and attempted rape were not known to the wider community. A female participant stated,

*Sexual violence does occur in our community, but people mostly do not report or disclose it, but they tend to keep it amongst themselves and their families.*

The participants explained the identity of the rape perpetrator and victim. They identified the perpetrator as a rich, influential, and relatively powerful man from the community. The victim was portrayed as a poor and isolated woman which lesser social ties. It was known from the discussions that most of the rape cases in the community were settled with financial negotiations and monetary compensations for the victim rather than finding legal remedies. It can be inferred that the victimization of women intersects with gender, wealth, social stature, and affluence. The participants feared that this practice of settlement of rape with money could make rape a commodity available for the powerful, rich, and affluent men to exploit and victimize women. A male participant clarifies,

*Recently, a man in his sixties raped a young girl near our village. The victim's family was ready to settle with monetary compensation offered by the rapist, but the involvement of the community stopped it and the rapist was handed over to the police.*

The participants shared available coping mechanisms against violence practiced in the community by women. It was learned that the victim of household violence mostly used community consultation and police reporting to evade further violence. They divulged that community consultation and police reporting resulted in decisions in favor of victim women, directing abusive husbands to show decency and stop committing violence. The fear of legal repercussions such as spending time in police custody and getting charged under domestic violence cases was understood as the reasons for husbands to stop abuse and violence. The discussions revealed that women who file a formal complaint about their husband’s violent behavior could face an increased risk of violence. The participants disclosed that sharing such incidents publicly brought shame to some of the men and increased their anger, and often backlashed with increased violence. The participants in both groups stated that not all women in the community reported violence. They identified that women tend to be quiet despite facing continuous violence due to the fear of encountering more violence and to keeping their families together. A female participant clarifies,

*Lodging public complaints against the abusive husband can sometimes escalate the violence. The husband’s anger for being humiliated in public must be faced by the woman inside the closed doors of the house with more violence and the men’s threat of abandoning the relationship.*

The participants stated that socio-cultural violence against women in dowry-related cases was widespread and increasing. The dowry exchange was explained as a traditional practice with the family of the bride paying cash and kind to the groom's family. The participants clarified that the practice of dowry in the earlier days must have been an emergency fund for the newly wedded bride in a newer setting. According to the participants, the system of dowry has now developed and evolved as a practice of forced involuntary transfer of goods and cash demanded by the groom’s family. The discussions disclosed that the demands for dowry were increasing with time and failing to provide as promised immediately resulted in violence for the newly wedded bride. The participants described that dowry-related violence starts with taunts and progresses to withholding of food, verbal abuse, and finally, physical violence. They added that perpetrators of such violence were both men and women from the groom’s family. They stated that due to poverty not all bride families in the community were able to supply all demanded dowry which has exposed a large number of women to face dowry-related abuse and violence. The discussions also informed of a newer trend among girls by demanding goods during their wedding. It was shared that this new emerging trend had increased a two-fold financial burden on the bride’s family with heavy marriage debts. The male participants when questioned about the dowry demands cunningly shifted the responsibilities towards family and stated that it was not the groom but their families who were making such dowry demands. The discussions verified that dowry practice was so engraved in the community that it was impossible to even imagine a marriage without any dowry. A male participant reflected,

*If I marry without any dowry, my family, neighbors, and all whom I know would consider that I am insane.*

The participants also discussed and identified harmful traditional practices present in the community. The participants informed a common practice of accusing women of as witches existed in the community. It was mentioned that women faced witchcraft allegations in different situations. They provided examples of witchcraft allegations in common situations such as when someone’s cow stops producing milk when a child has a sore eye, when someone is bedridden due to sickness for days, or when a woman undergoes a miscarriage, etc. The participants stated that women accused of witch were always elderly/single women living in seclusion, poverty, and with fewer social ties. They also shared that the witch doctors, who ascertain whether a woman is a witch or not, were surprisingly mostly always men and hold higher status, respect, and social recognition. The consequences of being labeled as a witch, as explained by the participants, haunted victim women with torture, name-calling, social boycott, and extremes of physical violence. The participants informed that inhumane practices such as forceful feeding of human excreta prevailed during the witch cleansing sessions. A female participant explaining the witchcraft situation stated,

*Witchcraft accusation is very real in our community; I know someone who has tortured his mother, citing reasons for his wife being childless. The old woman was called names, beaten, and later thrown out of the home.*

The participants felt that men’s use of violence and its legitimization primarily existed due to gender hierarchy and internalization of the belief that violence was the best method to resolve any conflict. They inferred that men’s use of violence was further reinforced by women's acceptance and belief that violence had occurred due to their faults and carelessness. The female participants shared examples of common household situations that could result in an episode of violence such as women cooking distasteful food, failing to provide timely care to children and the elderly due to workload, and forgetting to clean rooms. These incidents make women believe that violence majorly occurred due to their mistakes. Furthermore, the participants believed that this self-blaming of the victim resulted due to constant exposure to violence and a non-negotiable social positioning of women for raising questions. The participants stated that beliefs instilled by religion increased the likelihood of victimization for women. They explained that religious practices and ideologies required women to refer to their husbands as godly figures, and a religious belief that anything said or done against husbands was a disgrace bringing sin upon her and family positioned women in an inferior position. A male participant added,

*We belong to a culture where females worship their husbands as a god, and this might be an important reason for men to feel powerful as a god to exploit and abuse women.*

The discussions put forward the idea that the existence of discriminatory beliefs, reinforcement of such beliefs, and a blind following of such practices produced differences and violence. The male participants acknowledged that the idea of male supremacy not only produced violence but also established a belief system that considered violence as an indispensable way to treat deviated women. One male participant stated this idea of male supremacy and privilege as,

*The language of the feet is essential when words fail.*

The participants also discussed violence committed toward men by women. The male participants burst into laughter when they stated that some men were beaten by their wives when they were drunk. The male participants admitted that intoxication reduced their strength and they got beaten. The female participants, on the other hand, assumed that women hit intoxicated men due to frustration and helplessness. They further clarified that the act of husband beating was a situational reaction towards men who had spent all of their daily earnings on alcohol. They stated that women with the responsibility to cook and feed family find themselves in an utterly helpless situation by the irresponsible drinking behavior of men. The male participants shared incidences of violence against men due to foreign migration. It was revealed in the discussions that some of the migrating men’s wives had run away with remitted money, abandoning marriage, and breaking up the family. The male participants identified this as a form of victimization of men, furthermore, the spreading of rumors and gossip caused emotional instability in those men. The female participants confirmed that some returning men failed to find their homes, property, money, and/or their wives. The discussion participants in both groups identified that this practice was on the rise in the community. It became apparent from the discussions that this increasing trend of women running away with the money and breaking away from family was a personal issue requiring social remedies.

### Chasing dreams

The participants referred to sex trafficking as the exploitation of women, arising from poverty, illiteracy, and deceit. Explaining the causes of trafficking, the participants stated that women living in poverty, having dreams of prosperity and abundance were tricked by the traffickers making them victims of sex trafficking. The participants mentioned that women who had dreams larger than life and yearned for a comfortable and luxurious life in a short time were at a greater risk for sex trafficking. The participants from both groups resonated that the traffickers had been manipulating the dreams of poor women and deceiving them into trafficking. A female participant elaborated,

*Women in poverty can be fooled easily with dreams. She can be tricked by a trafficker by saying I will find you employment with good pay abroad, and she gets into the trap easily.*

A male participant further clarified,

*Women readily fall into fraud and trickery shown by the traffickers who assure of luxurious life with foreign employment and this bait often leads to sex trafficking.*

They identified that false hopes for foreign jobs were primarily used as an entry point by the traffickers to trap potential victims. Besides, they stated that some traffickers tricked women with false romantic relationships and marriages to win over their trust enabling traffickers to maneuver women as they wished.

It was identified that traffickers were not always strangers but known and familiar faces from the community, allowing the traffickers to gain the victim’s trust. The discussions divulged that traffickers strategically chose women who were less educated and poor. The participants explained that sex trafficking mostly occurred among women from a lower caste (the caste system is hierarchy-based in Hindu society which is determined by birth and unchangeable). They further explained that if one of these lower caste women went missing, it seldom raised any serious concerns in society, making these women easy targets for the traffickers. The discussions revealed that life for the survivors of sex trafficking was difficult. They identified that the survivor had to face strong stigmas and stereotypes which further increased their risk for re-victimization. The participants explained that the social acceptance of the trafficking survivors was minimal and finding a job for survival was very difficult. It was reported that social beliefs, norms, and practices were rigid for sex trafficking survivors and provided lesser opportunities for complete social integration. A female participant stated,

*The story of a sex-trafficked woman does not end after her rescue. It is difficult for her to live in society, and this increases her chances of being a further victim.*

The discussions in both groups highlighted that education and awareness were important for reducing sex trafficking. The participants felt that securing a livelihood for women was essential, but they identified it as a major challenge. The female participants recommended the use of education and awareness for reducing sex trafficking. They demanded effective legal actions and stringent enforcement of the law with maximum punishment for offending sex traffickers. They mentioned that the fear of law with maximum punishment for culprits could help decrease cases of trafficking.

### The theoretical concept of power play

The discussions identified that gender inequality and violence against women occurred as men possessed and exercised greater authority. The participants explained that the authority emerging from male-centric beliefs was reinforced through established socio-cultural institutions. It was known that oppressive practices toward women in both public and private life have led to the domination and devaluation of women. The differences between men and women were known to be instilled by evoking discriminatory beliefs and due to internalization of them as fundamental truths by women which further helps to sustain these created differences.

The concept of power-play developed from the study has its roots in the belief systems and was found constantly used by men to maintain created differences. The power-play rise due to patriarchy, guiding discriminatory norms and unequal gender practices. These norms and practices in the canopy of patriarchy positions women inferior to men and impose control and restrictions. The power play possessed multi-dimensional effects on women such as creating further barriers, restricted life opportunities, the need for men-centered anchoring systems, and exclusion from the public arena. The power play gains its strength from the strict enforcement of stereotypical practices and committed adherence to gender performances. This leads to internalization of subordination as a natural occurrence by women. These further isolate women putting them into several non-negotiating positions. The power play at an individual level provides restrictive movement for women, barring them from quality education and other life opportunities, and is exhibited in alcohol-related assault and sexual violence. At the structural level, this power play limits women from economic opportunities, access to resources, and decision-making, and induces socio-cultural inequality exhibited in dowry and cases of witchcraft. The socio-cultural acceptance of power-play allows men to use violence as a misuse of power and use it as an effort to maintain authority. The use of power-play for committing violence was identified as the worst display of exercised power play.

**Fig. 1:**
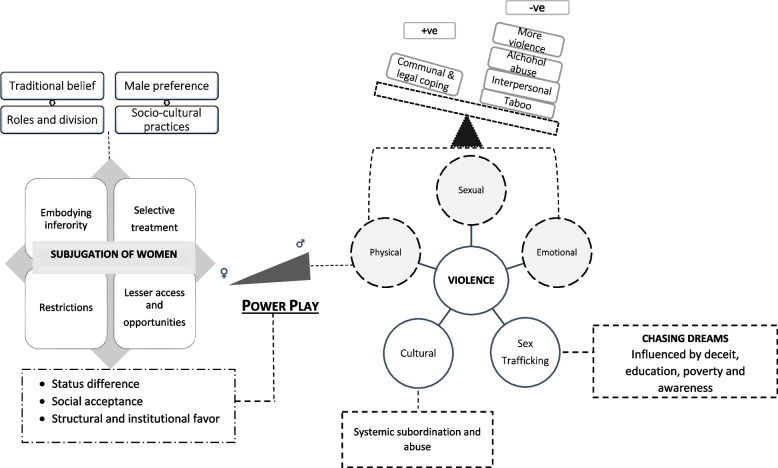
The theoretical concept of power play

Figure 1 describes the concept of power-play developed from the study. The power-play model is based on discussions and inferences made from data analysis. The model provides a description and explanation of how women are subjected to inequality and face violence. The concept of power play derives its strength from the subjugated status of women which are based on selective treatment, self-embodiment of inferiority, imposed restrictions and due to lesser life opportunities. The power play gain legitimacy through social approval of the status differences between men and women and through social systems and institutions majorly developed and favoring men. The status difference between men and women and its approval by developed social institutions and processes give rise to the concept of powerplay. It identifies that status differences allow men to gain and (mis)use power play not only to maintain differences but also enable men to use violence. The use of power-play exists at both interpersonal and cultural levels. Further, the model elaborates on influencers causing subjugation of women, display of power-play, and violence. The model identified that lodging public complaints and seeking legal remedies are the influencers that suppress violence against women. The influence of Forum Theater was perceived to have greater influence for victim, perpetrator, and bystanders. The influencers that aggravate violence are fear of further violence, the nature of the interpersonal relationship, alcohol-related abuse, and remaining silent especially on sexual violence. The cultural violence mentioned in the model refers to dowry and witchcraft-related violence and stands as systemic subordination. In the model, sex trafficking is depicted as one of the outcomes of inequality and violence faced by women majorly occurring due to deceit and fraud.

## Discussion

The theoretical concept of power-play developed in this study identifies that inequality produces violence and violence further reinforces inequality, creating a vicious circle. The power play situates hierarchy based on gender as the primary cause and identifies violence as an outcome of this power asymmetry. The authority to use power by men is received by social approval from embedded structures and institutions. The functioning of associated structures and norms is designed and run by men helping to perpetuate the dominance and subjugation of women. The study identifies that both interpersonal and socio-cultural violence emerges due to the positional differences and use of power. The study found that an element of control exists in interpersonal violence. The findings show that few victim women in the community took advantage of consultations and rely on the law to evade and /or cope during the occurrence of interpersonal violence. A large number of victims women however suffer silently as they are unable and unwilling to take a stand on violence due to their perceived positional differences and strict norms following. The study finds that violence originating from socio-cultural systems is widely accepted and no established means of control exists. The practice of heinous acts against a fellow human during witchcraft allegations and dowry exchanges is prohibited by the law of Nepal but is widespread. This situates that practices which are based on belief systems are more effective than prevailing national laws which try to stop them. Sex trafficking as a form of sexual violence use deceit and fraud against women. Poverty and illiteracy compel women to search for alternatives, and they become easy victims of sex trafficking when their dreams of a better life are manipulated by the traffickers. The false promise of a better life and highly paid job put women in a non-negotiating position with traffickers. The cherished dream of escaping the prevailing status-quo of oppression, subordination, violence, and poverty mesmerizes women to take risky decisions, falling into the risk and trap of sex trafficking.

The socio-cultural norms are the unwritten script of social operatives and functioning. These social norms function as codes of operation and are a major determinant for behavior and interactions between people [43]. The study has found that these norms were skewed, and most favored men, giving rise to status differences and producing inequalities for women. This is observed with lesser life opportunities, lower participation in decision-making, and a constant need to anchor women. This further helps men to maintain their hierarchical positional status and use violence. The subjugation of women does not occur in a linear process, it is influenced by the internalization of discrimination resulting in lower self-esteem, suppression, and domination of women based on norms and unequal practices. Earlier research has identified that norms and beliefs encourage men to control women, and direct them to use force to discipline women which increases the risk of violence occurrence [44, 45]. An earlier study shows that traits of masculinity require men to become controlling, aggressive, and dominant over women to maintain status differences [46]. The study confirms that men upon receiving both normative and social approval for using violence against women can do so without hesitation.

Violence against women in Nepal mostly occurs inside the home and is only reported when it reaches higher levels of severity. The acceptance of violence as a private affair has restricted women from seeking support and discourages them from communicating their problems with outsiders [47] this increases more likelihood for men to use violence. The study finds issues related to sex and sexual violence is a taboo and are seldom reported. The study could only identify cases of sexual assault registered with the police and other cases known to the wider community as sexual violence. A community with known incidents of rape may have other cases of abuse, harassment, incest, forceful sexual contact, etc. Failure to report incidents of sexual violence infer that a large number of women could be suffering in silence. Earlier research identifies that increased stigmatization associated with sexual violence, and fear of seclusion cause reluctance in victims to report or seek support [48]. This silencing of victims provides men with greater sexual control over women [49] increasing more likelihood of use of violence. Gender-based inequality and violence intersect structures, institutions, and socio-cultural processes, making inequality and violence visible at all levels. The dowry-related violence and witchcraft allegation intersect interpersonal and structural violence. This cultural violence forces women to be a victim of lifelong abuse and trauma. The intersecting relationship between gender norms, social structures, and individual is so closely knitted that it produces varieties of inequality and violence at all levels [50]. Emotional violence in this study only emerged as a type of violence, during discussions in both groups. It did not emerge as a major concern for the participants except for dowry-related violence and violence against men. The intertwined nature of emotional violence and its occurrence with each abusive, exploitative, and violent situation may have influenced the participants understand it as a result, rather than as a specific type of violence.

The power play between sexes was found in synchronicity with the established norms and prevailing stereotypes, helping to perpetuate gender power imbalance. The gender system is influenced and governed by norms and the social arena becomes the site of its reproduction through the interaction and engagement of people. This interaction provides approval to the institutions and processes that are based on constructed differences between men and women [51]. The power, as identified by Fricker [52], controls a social group and operates and operates through the agent or established social structures. A man can actively use the vested power to either patronize and/or abuse women while passively women’s internalization of social settings and embedded norms can put them docile. The social controls as reported by Foucault [53] work with the embedded systems of internalization, discipline, and social monitoring and uses coercion rather than inflicting pain. The internalization of status differences among women as indicated by the study confirms this schema of social control. The dominance of men over women with patriarchal beliefs establishes the significance of male-centered kinship. This requires women to constantly anchor with men providing grounds for inequalities to perpetuate further. This idealizes men and reinforces the belief that women are non-existent without their presence. The requirement for male anchorage has an attachment to prevailing structural inequality. The family property and resources are mostly controlled by men and it usually transfers from father to son limiting inheritance to women [51]. These glorified idealizations of men's competence as described by Ridgeway [54] idealize men as individuals with abilities, status, power, and influences. The need for women to rely on men as anchors, fear of going against the norms and social sanctions explains the positional difference and show that men possess greater competencies. The internalization of men-centric superior beliefs by women occurs due to self-passivity and devalues women creating false impressions of their abilities. The gender roles and responsibilities were strict for both sexes but provided greater flexibility, privilege, and opportunity for men. Earlier studies in congruence with this study find that socio-cultural expectations limit women from deviation, and strictly adhere to their prescribed role and expectations [55, 56] providing an upper hand to the men. The unequal social positioning of women, as defined by a few of the participants, can help define men's use of violence. As inferred by Kaufman [57], the disadvantageous position of women and support from the established structures enable men to use aggression and violence with considerable ease. The concept of power-play derived from this study also reflects that inequalities not only create hierarchies, putting women into a subordinating position but also legitimize norms of harmful masculinity and violence [57–60] creating a vicious cycle of inequality and violence. The concept of power-play developed by this study requires further exploration of gender relations, injustice, and patriarchy to identify multiple operatives of power with an outcome of inequality and violence.

## Strengths and limitations of the study

The study followed the same participants over a period, which helped the study to achieve clarity on the topics through constant engagement. The data collection and the initial data analysis of the study were conducted by the same person, which reduced the risk of misrepresented findings. The study used follow-up discussions, which provided an opportunity to meet the participants again to resolve any ambiguities. The constant engagement with the participants helped to develop rapport and trust, which is essential to enable meaningful discussions. The study gathered rich data for developing the theory of power play in the Nepalese context. The study has attempted to explain the interplay of men’s use of power play, gender inequality, and violence against women, which, in itself, is a complex, but important issue. The study helped to develop a platform by identifying a level of awareness and needs for a Forum Theatre intervention study, a first of its kind in Nepal.

The major limitation of the study is that it was conducted with only one of the ethnic populations of Nepal; thus, the findings from this study cannot be generalized to a completely different setting. However, the transferability of the study is possible in a similar setting. The incidences of inequality and violence shared by the participants were self-reported, and no other means of verification were available to crosscheck those claims. The differences among the participants both in and between groups based on education and marital status might have influenced the study participants to understand, observe, and experience the phenomenon. The possibility of social desirability bias remains with the study, as a constant engagement with the study participants might have influenced them to answer differently. Furthermore, the discussions were conducted in groups, and participants might have had hesitation to bring up any opposing views. The study relied on collecting information on social norms and individual experiences and the perceptions of the study participants. It cannot be claimed that the study is devoid of any data rigidity as participants were free to choose what they wanted to share and express.

## Study implications

The study explains gender practices, norms, violence against women, and sex trafficking in Nepal. The study helps to increase the understanding of how gender systems are operative in the daily lives of the Tharu community in the Morang district of Nepal. Future studies can explore the established linkages of interpersonal and socio-cultural violence. Like the complex link existing between gender inequality and violence against women, interpersonal violence and socio-cultural violence cannot be studied in isolation. The study provides an opportunity for future research on exploring how changing norms have been altering the position and victimization of women. The study finds that changing gender norms and responsibilities have, on the one hand, provided agency and empowerment for women, but on the other hand, they have also increased their risk of being a victim, an area that requires further exploration. The study has identified that constant engagement with the study participants through follow-up studies ensures the richness of data, which can be useful information for a future research study design. The study can be helpful for policy development, social activists, leaders, and researchers as it discusses prevalent gender oppressions and victimization, which need to be addressed. The findings from the study can be helpful for dialogue imitation and for designing intervention projects aimed at providing justice and equality to women.

## Conclusion

The study identifies the presence of gender inequalities and violence against women in the study area. The positional differences based on norms, institutions, and practices have assigned greater privileges to men. The concept of power-play devised by the study ascertains the maintenance of gender hierarchy to produce inequality further and victimization of women. The subjugation of women based on the social-cultural process, embedded belief systems, and norms prevent women from life opportunities and dignified life. It situates men at the highest rung of the gender and social ladder providing a comparative advantage for men to use power. Violence emerges as men’s use of power play and as a strategy for the continued subjugation of women. Sex trafficking as a consequence of inequality and violence has its origins in illiteracy and poverty with women falling prey to the deceit of traffickers. It is important that dreams for progression provide motivation for women to develop further but at the same time, dreams should not be exchanged with trickery and fraud offered by the traffickers. Awareness and attitudinal changes are imperative to challenge unequal norms, and practices, and reduce the risks of sex trafficking. This can help to develop negotiations for power-sharing which helps to reduce inequality, violence, and preparedness in chasing dreams. Changes at both individual and societal levels are necessary to develop a collective action for establishing belief systems and practices providing women with an equal position and reducing the risk of violence.

## Data Availability

The datasets generated and/or analyzed during the current study are not publicly available due to privacy but are available from the corresponding author upon reasonable request.

## References

[1] Felson RB, Outlaw MC (2007). The control motive and marital violence. Violence Vict.

[2] Wamala S, Ågren G. Gender inequity and public health: Getting down to real issues. European Journal of Public Health. 2002. 10.1093/eurpub/12.3.163 12232952

[3] World Health Organization. Promoting gender equality to prevent violence against women. World Health Organization. 2009.

[4] Devries K, Maki J, Garcia-Moreno C, Petzold M, Child J, Falder G (2013). The Global Prevalence of Intimate Partner Violence Against Women. Science (80- ).

[5] Walby S. Theorising patriarchy. Sociology. 1989;

[6] Butler J. Undoing gender. Routledge. 2004.

[7] Yllo K (2005). Through a Feminist Lens: Gender, Diversity, and Violence: Extending the Feminist Framework In Current Controversies on Family Violence.

[8] Heise L (1998). Violence against women: An integrated, ecological framework. Violence Against Women.

[9] Pulerwitz J, Barker G (2008). Measuring attitudes toward gender norms among young men in Brazil: Development and psychometric evaluation of the GEM Scale. Men Masculinities.

[10] Jakobsen H (2014). What’s Gendered about Gender-Based Violence?: An Empirically Grounded Theoretical Exploration from Tanzania. Gend Soc [Internet].

[11] Cook RJ. Gender, Health and Human Rights. Heal Hum Rights Quart. 1995;350–68. 10393793

[12] Freeman MA. Reservations to CEDAW: An Analysis for UNICEF. Discussion Pper [Internet]. UNICEF; 2009. Available from: http://www.unicef.org/gender/files/Reservations_to_CEDAW-an_Analysis_for_UNICEF.pdf

[13] UNIFEM (2007). Facts and figures: Violence against women [Internet].

[14] WHO (2005). Multi-country study on women’s health and violence against women [Internet].

[15] UN Women. World Survey on the Role of Women in Development. Gender Equality and Sustainable Development. New York: United Nations; 2014.

[16] Thomas D, Beasley M (1995). Domestic Violence as a Human Rights Issue. Albany Law Rev.

[17] Coomaraswamy R. Special Rapporteur on Violence against Women, its Causes and Consequences, Integration of the Human Rights of Women and the Gender Perspective: Violence U.N. Doc. E/CN.4/1999/68 Against Women: Violence against women in the family [Internet]. Geneva, Switzerland; 2002. Available from: https://digitallibrary.un.org/record/459009?ln=en

[18] OHCHR. 15 Years of the United Nations Special Rapporteur on Violence against Women (1994–2009)-A Critical Review [Internet]. Office of the United Nations High Commissioner for Human Rights (OHCHR); 2009. Available from: https://www.ohchr.org/Documents/Issues/Women/15YearReviewofVAWMandate.pdf

[19] Stephanie Rose M (2015). The role of structural and interpersonal violence in the lives of women: a conceptual shift in prevention of gender-based violence. BMC Women Heal.

[20] Farmer PE, Nizeye B, Stulac S, Keshavjee S (2006). Structural violence and clinical medicine. Plos Med.

[21] WHO (2002). World Report on Violence and Health.

[22] Connell RW. Hegemonic Masculinity. Jackson S, Scott S, editors. Gender: A Sociological Reader. London: Routledge; 1987.

[23] UNFPA. Overcoming violence against women through an integrated and community-based approach Vol. 2, Programming to Address Violence Against Women. 8 Case Studies. 2008.

[24] Pigg S (1992). Inventing Social Categories through Place: Social Representations and Development in Nepal. Comp Stud Soc Hist.

[25] Lamichhane P, Puri M, Tamang J, Dulal B (2011). Women’s Status and Violence against Young Married Women in Rural Nepal. BMC Womens Health.

[26] Atteraya MS, Gnawali S, Song IH (2015). Factors Associated With Intimate Partner Violence Against Married Women in Nepal. J Interpers Violence.

[27] Ministry of Health N, New Era. Nepal Demographic and Health Survey 2016 [Internet]. Kathmandu, Nepal: MOH/Nepal, New ERA, and ICF; 2017. Available from: http://dhsprogram.com/pubs/pdf/FR336/FR336.pdf

[28] Ghimire DJ, Axinn WG, Smith-Greenaway E. Impact of the spread of mass education on married women’s experience with domestic violence. Soc Sci Res. 2015; 10.1016/j.ssresearch.2015.08.004 PMC4607934 26463551

[29] Joshi S, Kharel J. Violence against Women in Nepal -- An Overview. Free Libr. 2008;

[30] Government of Nepal. A study on gender based violence conducted in selected rural districts of Nepal. Kathmandu, Nepal: Office of the Prime Minister and Council of Ministers; 2012.

[31] Deuba K, Mainali A, Alvesson HM, Karki DK (2016). Experience ofintimate partner violence among young pregnant women in urban slums of Kathmandu Valley, Nepal: a qualitative study. BMC Women’s Heal.

[32] Sharma S, SRIF/SNV. Domestic violence in Nepali society: root cause and consequences a research report. Social Inclusion Research Fund; 2007.

[33] Joshi SK (2009). Violence Against Women (VAW) in Nepal: Role of Health Care Workers. Kathmandu Univ Med J.

[34] Dahal P, Kumar Joshi S, Swahnberg K (2015). “We are looked down upon and rejected socially”: A qualitative study on the experiences of trafficking survivors in nepal. Glob Health Action.

[35] Huda S (2006). Sex trafficking in South Asia. Int J Gynecol Obstet.

[36] Hendrickson ZM, Owczarzak J, Lohani S, Thapaliya Shrestha B, Underwood CR (2019). The (re)productive work of labour migration: the reproductive lives of women with an absent spouse in the central hill region of Nepal. Cult Heal Sex.

[37] Hendrickson ZM, Lohani S, Thapaliya Shrestha B, Underwood CR (2018). Talking about reproduction with a migrating spouse: Women’s experiences in Dhading. Nepal. Health Care Women Int.

[38] DDC. District Profile Morang 2070 [Internet]. Biratnagar, Morang; 2013. Available from: http://ddcmorang.gov.np/wp-content/uploads/2016/04/District-profile-2070.pdf

[39] Dahal P, Joshi SK, Swahnberg K. Does Forum Theater Help Reduce Gender Inequalities and Violence? Findings From Nepal. J Interpers Violence [Internet]. 2021 Mar 4;0886260521997457. Available from: 10.1177/0886260521997457 10.1177/0886260521997457 PMC9251741 33663256

[40] Dahal P, Joshi SK, Swahnberg K. The Prevalence of gender inequalities and gender based violence in eastern Nepal. Kathmandu Univ Med J. 2019;17(4 [ISSUE 68|OCT.-DEC). 33311039

[41] Hummelvol JK. The Multistage Focus Group Interview A Relevant and Fruitful Method in Action Research Based on a Co-operative Inquiry Perspective. Nor Tidsskr Sykepl. 2008;10.

[42] Chamraz K (2010). Constructing Grounded Theory A Practical Guide Through Qualitative Analysis.

[43] Darlauf SN, Blume LE (2008). New Palgrave Dictionary of Economics.

[44] Ilika AL (2005). Women’s Perception of Partner Violence in a Rural Igbo Community. Afr J Reprod Health.

[45] Mitra A, Singh P (2007). Human Capital Attainment and Gender Empowerment: The Kerela Paradox. Soc Sci Q.

[46] Niaz U (2003). Violence against women in South Asian countries. Arch Women’s Ment Heal.

[47] Khan H (2008). Women’s perceptions and experiences of sexual violence in marital relationships and its effect on reproductive health. Health Care Women Int.

[48] Sable M, Danis F, Mauzy D, Gallagher SK (2006). Barriers to reporting sexual assault for women and men: perspectives of college students. J Am Coll Heal.

[49] Flood M, Pease B (2009). Factors Influencing Attitudes To Violence Against Women. Trauma Violence Abuse.

[50] Jewkes R, Penn KL, Rose JH (2005). ‘‘If they rape me, I can’t blame them”: Reflections on gender in the social context of child rape in South Africa and Namibia. Soc Sci Med.

[51] Cislaghi B, Heise L (2019). Using social norms theory for health promotion in low-income countries. Heal Promot Int.

[52] Fricker M. Epistemic Injustice: Power and the Ethics of Knowing. Oxford: Oxford University Press; 2007.

[53] Foucault M (1995). Discipline and Punish: The Birth of the Prison.

[54] Ridgeway CL (2009). Framed Before We Know It: How Gender Shapes Social Relations. Gend Soc.

[55] Hollander J (2013). Demand More of People: Accountability, Interaction, and Gender Change. Gend Soc.

[56] West C, Zimmerman D (1987). Doing Gender. Gend Soc.

[57] Kaufman M (1987). The Construction of Masculinity and the Triad of Men’s Violence. Beyond Patriarchy: Essays by Men on Pleasure, Power, and Change.

[58] Connell R (1995). Masculinities.

[59] Courtenay WH (2000). Constructions of masculinity and their influence on men’s well-being: a theory of gender and health. Soc Sci Med [Internet].

[60] Jakobsen H (2014). What’s Gendered about Gender-Based Violence?. Gend Soc.

